# Long-term control of large pontine arteriovenous malformation using gamma knife therapy: a review with illustrative case

**DOI:** 10.1002/brb3.149

**Published:** 2013-06-12

**Authors:** Martin M Mortazavi, Daxa Patel, Christoph J Griessenauer, R Shane Tubbs, Winfield S Fisher

**Affiliations:** 1 Division of Neurosurgery, Department of Surgery, University of Alabama at BirminghamBirmingham, Alabama; 2 Pediatric Neurosurgery, Children's HospitalBirmingham, Alabama

**Keywords:** Arteriovenous malformation, brain stem

## Abstract

Brain stem arteriovenous malformations (AVMs) are rare and their clinical management is controversial. A location in highly eloquent areas and a greater risk of radionecrosis are both serious issues for radiosurgery of this entity. We report a case of a pontine AVM treated successfully with gamma knife therapy. At 3 years angiographic follow-up, imaging demonstrated complete thrombosis and there were no new neurological deficits, and at 7 years clinical follow-up, the patient continued to be neurologically stable. Although all treatments carry risk of neurological compromise, gamma knife therapy may offer the best treatment option for brain stem AVMs as seen in the case presented herein. This case illustrates a rare case of holo-pontine AVM tolerating gamma radiation with complete angiographical response and minimal neurological sequalae.

## Introduction

Arteriovenous malformations (AVM) are congenital vascular malformations with direct arterial to venous connections without an intervening capillary network (Doppman 1971). The abrupt transition from a high-pressure arterial system to a low-pressure venous system leads to venous engorgement with subsequent arterialization of the venous limb, resulting in edema and irritation of surrounding brain tissue. This predisposes the patient to bleeding with or without associated arterial and/or venous aneurysms (Houdart et al. 1993; Miyachi et al. 1993; Valavanis 1996). Therefore, intracranial AVMs usually present with hemorrhage, seizures, headache, and focal neurological deficits (Crawford et al. 1986; Brown et al. 1996; Mast et al. 1997). Seizures and neurological deficits are secondary to mass effect or steal phenomenon. Brain AVMs occur in about 0.1% of the population, accounting for 3% of strokes and 9% of subarachnoid hemorrhages (Drake et al. 1986; Schauble et al. 2004; Maruyama et al. 2005). The risk of bleeding is 2–4% per year and the average annual mortality from untreated AVMs is 1.0% (Brown et al. 1988; Ondra et al. 1990; Stapf et al. 2006; da Costa et al. 2009). In one report, the annual hemorrhage rates ranged from 0.9% for patients without hemorrhagic AVM presentation, deep AVM location, or deep venous drainage to as high as 34.4% for those harboring all three risk factors (Stapf et al. 2006).

The main diagnostic tools for these pathologic entities are magnetic resonance imaging (MRI), CT angiogram, and angiography (Al-Shahi and Warlow 2001). Surgery and radiosurgery are the treatments of choice depending on the size and location of the AVM. Endovascular embolization is only considered as an adjunct as embolization alone leads to relatively rapid vessel recruitment (Friedlander 2007). The original 5-tier Spetzler–Martin classification and the recent 3-tier modification of this system have provided a practical tool in terms of surgical risks and outcomes (Spetzler and Martin 1986). Low grades are amenable to surgical resection, higher grades are usually not candidates for surgery, and grade IIIs (group C in the newly proposed classification) require a multimodal approach (Spetzler and Martin 1986). Lack of definitive treatment strategies for high-grade AVMs has led to modified radiosurgical strategies.

Generally, complete obliteration of the AVM with radiosurgery depends on the size of the lesion and the maximum without deficit dose of radiation (Ondra et al. 1990; Fabrikant et al. 1992). One series reported an 80% response rate to radiation at 3 years for lesions that were 3 cm or smaller (Ondra et al. 1990; Pollock and Meyer 2004). Even with larger AVMs, some amount of lesion reduction occurs and additional treatment is effective in most (Foote et al. 2003; Pollock and Meyer 2004). Flickinger et al. (1996) reported a 72% overall obliteration rate in a retrospective series of 197 patients receiving radiosurgery. A larger series of 1319 patients from the Karolinska Institute reported by Karlsson et al. (1997) found an 80% overall obliteration rate. Furthermore, the authors reported the chance of obliteration being ∼90%, 80%, and 70% for AVMs given isodoses of 20 Gy, 18 Gy, and 16 Gy, respectively.

The risk of radiation-associated complications is related to the location of the AVM, AVM volume, and radiation dose. For larger AVM volumes, the radiation dose is typically decreased so that the chance of radiation-related complications is <5%. Other groups have reported comparable results showing complications of stereotactic radiation are due to AVM location and the total volume of treatment (Flickinger et al. 2000; Skjoth-Rasmussen et al. 2010). Deeper locations like the thalamus, basal ganglia, or brain stem and larger volumes of therapy carry greater risks of deficits (Miyawaki et al. 1999; Flickinger et al. 2000). Most studies have documented an ∼2–3% risk of radiation necrosis with permanent neurologic deficits (Fabrikant et al. 1992; Pollock and Meyer 2004). Therefore, in an eloquent location such as the brain stem, even radiosurgery carries significant risks.

Another disadvantage of radiosurgery compared to surgical resection is that patients continue to have hemorrhage risk until the AVM is completely obliterated. Karlsson et al. reported the latency interval from radiosurgery to obliteration as lasting between 1 and 4 years. There is conflicting evidence regarding the risk of hemorrhage during the latency period. Steinberg et al. in 1990 and Fabrikant et al. in 1992 reported an increased risk during this time. However, from a cohort of 500 patients, Schauble et al. presented strong evidence supporting a reduced risk of hemorrhage during the latency period (Maruyama et al. 2005). Improved seizure control may be an added benefit of radiation (Schauble et al. 2004).

Approximately 10% of AVMs are located in the posterior cranial fossa and the prognosis is poor for patients with AVMs in this area (Drake et al. 1986). In 1986, Drake et al. reported a series of 66 surgically treated AVMs including ponto-medullary AVMs. Seven of these eight AVMs were <2.5 cm in diameter and one was ∼5 cm in diameter. Of these eight, one patient died after exploration, and two patients had poor outcomes (Drake et al. 1986). Microsurgical resection of these deep AVMs leads to greater mortality and decreased rates of complete resection (Drake et al. 1986; Massager et al. 2000). Embolization has not been used as the sole treatment of brain stem AVMs although there is no long-term analysis or randomized clinical trials (Duma et al. 1993; Kurita et al. 2000; Massager et al. 2000), previous studies document that the most efficacious and safest mode of treatment for brain stem AVMs is modified stereotactic radiosurgery (Flickinger 1989; Lunsford et al. 1991; Flickinger et al. 1992, 2000, 2002; Duma et al. 1993; Pollock et al. 1996, 1998; Karlsson et al. 1997; Kurita et al. 2000; Massager et al. 2000; Bhatnagar et al. 2001; Hadjipanayis et al. 2001; Pollock and Flickinger 2002). The Flickinger study and the larger Karlsson study mentioned earlier, did not report any numbers for the gamma knife outcome on the brain stem AVMs. This case report follows the course of a patient with a large brain stem AVM that was completely eradicated with gamma knife therapy.

## Case Report

**Figure 1:**
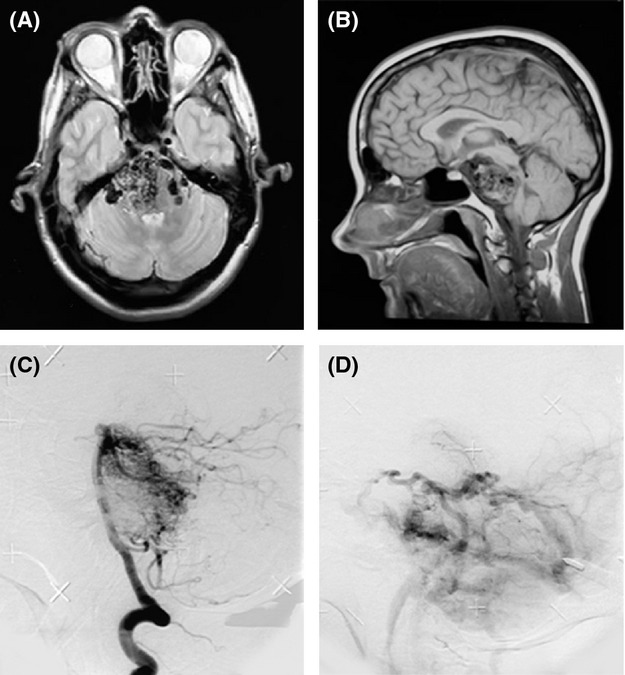
AVM located in right pons. (A) Axial T2-weighted MRI brain. (B) Sagittal T1-weighted MRI brain. (C) Digital subtraction arteriogram, vertebral artery injection, lateral view, arterial phase. (D) Digital subtraction arteriogram, vertebral artery injection, lateral view, venous phase. AVM, arteriovenous malformations; MRI, magnetic resonance imaging.

**Figure 2:**
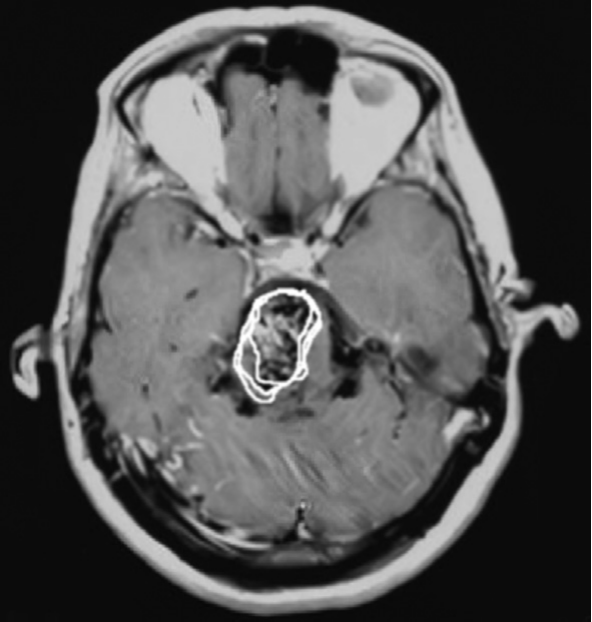
Gamma knife dosimetry and treatment plan.

**Figure 3:**
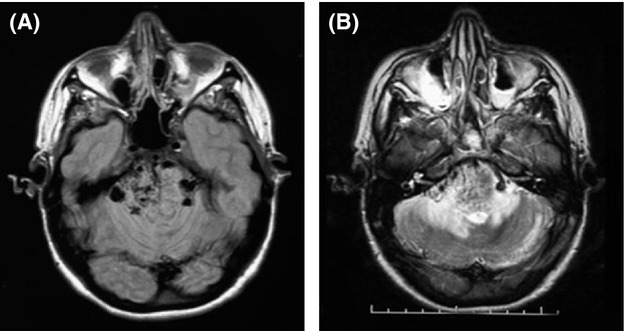
AVM located in right pons after gamma knife treatment. (A) Axial T1-weighted MRI brain. (B) Axial T2-weighted MRI brain. AVM, arteriovenous malformations; MRI, magnetic resonance imaging.

**Figure 4:**
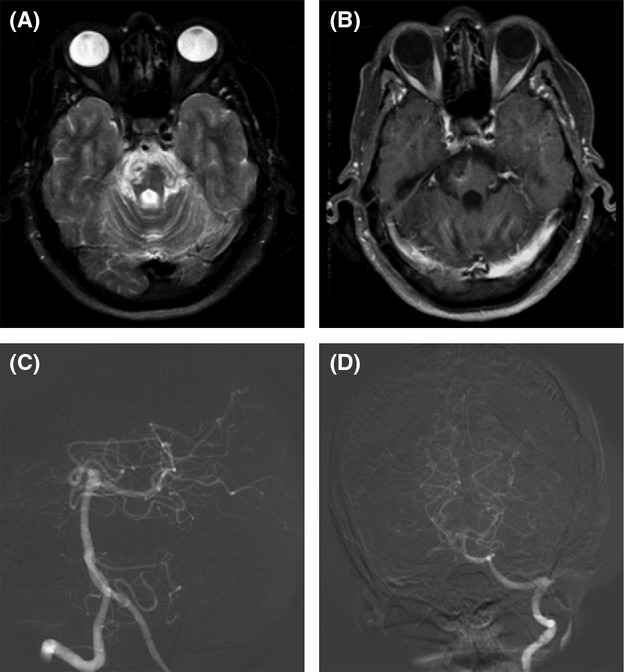
AVM located in right pons after gamma knife treatment. (A) Axial T2-weighted MRI brain. (B) Axial T1-weighted MRI brain with contrast. (C) Digital subtraction arteriogram, vertebral artery injection, lateral view, arterial phase. (D) Digital subtraction arteriogram, vertebral artery injection, anterior–posterior view, arterial phase. AVM, arteriovenous malformations; MRI, magnetic resonance imaging.

A 37-year-old right-handed white female presented in 1997 with a 2-year history of progressive left hemiparesis, ataxia, facial pain, and tongue numbness. Her physical exam revealed a mild left facial nerve palsy, and decreased light touch and pin prick on the entire left side. Imaging showed a 2.2 cm AVM centered in the right pons, supplied by branches of the basilar and right vertebral arteries (Fig. 1A–D). Additionally, there was significant dilation of both basal veins of Rosenthal and to a lesser extent, the vein of Galen and straight sinus (Fig. 1C). Due to worsening neurologic deficits and severe uncontrollable pain, the patient elected to proceed with gamma knife treatment in August of 1997. The total dose given to the 50th% was 17.5 Gy and the total volume was 1.49 cm^3^ (Fig. 2). Subsequently, the patient returned to clinic in February of 1998 complaining of increasing left hemiparesis, right upper extremity paresthesias, and falling. Neurologically, the patient was found to have a hemiparetic gait, left facial nerve palsy, left hemiparesis (4/5), and decreased light touch and pin prick on the left side. She was hyperreflexive on the left side. MRI showed significant evidence of edema in the right pons, cerebellum, and right basal ganglia and a reduction in the flow void signal of the AVM, with partial thrombosis of the large pontomesencephalic draining vein (Fig. 3A and B). The patient was admitted for hydration and intravenous steroid infusion. The patient's left hemiparesis persisted. She was continued on steroids, transferred for inpatient rehabilitation therapy, and then discharged home with outpatient physical therapy. The patient was followed annually with CT angiogram and MRI with and without contrast until 2004. She continues to have a mild left hemiparesis but her suicidal facial pain syndrome had resolved. MRI confirmed a partially calcified right pontine lesion with surrounding enhancement representing AVM with previous hemorrhage. At last angiographic follow-up 3 years after treatment, angiography supported eradication and complete thrombosis of the AVM in the right pons with no major feeding vessels or draining veins and apparent adjacent encephalomalacia (Fig. 4A–D).

## Discussion

As the first description in 1895, the treatment of cranial AVMs has been a topic of controversy. In the early 1980s, Spetzler and Martin introduced a 5-tier system in order to translate radiological findings to surgical risks and outcomes. This system was well accepted because of its simplicity and practicality. However, one important dilemma was the grade III AVM. As a small deep AVM in an eloquent area has the same grade as a large superficial AVM in a noneloquent area, the treatment options of this group cannot naturally be the same. The deep AVM group has therefore been extensively explored in search for the best treatment paradigm. In order to further simplify the grading system, in 2010, Spetzler and Ponce (2011) proposed a 3-tier grading system where grades I and II were put together as grade A, III renamed as grade B, and IV and V were combined as grade C. Comparison of the outcomes according to the new proposed system showed insignificant differences in risks and outcomes between the previous groups I through II and IV through V. Surgical resection was proposed for group A, multimodal treatment was proposed for group B, and observation with some exceptions was suggested for group C.

Brain stem AVMs are automatically classified at least as grade III in the old system and as grade B in the new system because they are always in eloquent brain and have deep venous drainage. Therefore, surgical resection rarely if ever leads to a good outcome. This is highlighted by the surgical series performed by Drake et al. and published in 1986. Endovascular embolization does not have a place in the armamentarium of brain stem AVMs, not only because new vessels continue to be recruited after the initial embolization, but also in light of the fact that the feeding vessels of the AVM usually have some involvement in the surrounding eloquent brain stem. Radiosurgery has appeared to be the only option, especially for grades IV through V in the old system vis-a-vis grade C in the new system. Overall, Flickinger reported a 72% and Karlsson reported an 80% overall response rate using gamma knife. However, none of these reports included separate reports on subgroups involving only brain stem AVMs and their outcome and radionecrosis rates.

The success rate of obliteration is proportional to the isodose. However, radiosurgery to brain stem AVMs offers serious considerations due to the risk of radionecrosis. The overall risk of radionecrosis is estimated to be 2–3% given the fact that lower isodoses are delivered to eloquent areas leading to less obliteration responses in these cases. Pontine AVMs offer treatment dilemmas as even low isodoses are associated with a high risk of radionecrosis while the obliteration rate is lower secondary to the low isodoses. As the pontine AVM increases in size, it is apparent that the risk of neurological compromise by the AVM itself increases along with a decreased chance of obliteration since higher isodoses cannot be freely delivered to the brain stem. Therefore, even small pontine AVMs have primarily been followed with observation.

Here, we report a case with a grade III (B according to the new proposed system), where single treatment of a pontine AVM with overlapping isodoses led to complete obliteration without any new neurological deficit.

## Conclusions

Any treatment of brain stem AVMs offers considerable risk for neurological compromise. Radiosurgery in highly selected cases may offer a treatment option with reasonable risks.
